# Tracking the Workforce 2020-2030: Making the Case for a Cancer Workforce Registry

**DOI:** 10.1200/GO.21.00093

**Published:** 2021-06-17

**Authors:** Archita Srivastava, Matthew Jalink, Fabio Ynoe de Moraes, Christopher M. Booth, Scott R. Berry, Fidel Rubagumya, Felipe Roitberg, Manju Sengar, Nazik Hammad

**Affiliations:** ^1^ Department of Medicine, Queen’s University, Kingston, ON, Canada; ^2^ Department of Oncology, Queen’s University, Kingston, ON, Canada; ^3^ Department of Oncology, Rwanda Military Hospital, Kigali, Rwanda; ^4^ Department of Oncology, University of Sao Paulo, Sao Paulo, Brazil; ^5^ Department of Medical Oncology, Tata Memorial Centre, Homi Bhabha National University, Mumbai, India

## Abstract

Existing literature has described the projected increase in cancer incidence and the associated deficiencies in the cancer workforce. However, there is currently a lack of research into the necessary policy and planning steps that can be taken to mitigate this issue. Herein, we review current literature in this space and highlight the importance of implementing oncology workforce registries. We propose the establishment of cancer workforce registries using the *WHO Minimum Data Set for Health Workforce Registry* by adapting the data set to suit the multidisciplinary nature of the cancer workforce. The cancer workforce registry will track the trends of the workforce, so that evidence can drive decisions at the policy level. The oncology community needs to develop and optimize methods to collect information for these registries. National cancer societies are likely to continue to lead such efforts, but ministries of health, licensing bodies, and academic institutions should contribute and collaborate.

## POLICY ISSUE

The WHO has identified cancer as one of the five noncommunicable diseases (NCDs) that in total are responsible for 71% of all deaths worldwide.^1^ Between 2008 and 2030, there will be an estimated 80% increase in the number of new cancer cases in low-middle–income countries (LMICs) and a 40% increase in new cases in high-income countries.^2^ This will be accompanied by a 45% increase in the number of global cancer deaths during the same period.^2^ It is therefore not surprising that NCDs are a prominent focus of Goal 3 of the United Nations (UN) Sustainable Development Goals (SDGs), which aims to reduce premature mortality because of NCDs by one third through prevention and treatment by 2030.^3^ An essential step in meeting Goal 3 is to address Target 3c: “Substantially increase health financing and the recruitment, development, training and retention of the health workforce in developing countries, especially in least developed countries and small island developing states.”^4^ WHO has introduced global milestones for health workforce to promote equitable access to health care workers to further progress toward achieving SDGs Target 3c.^5^

**CONTEXT**

**Key Objective**
To describe the role of tracking the workforce in generating policy and planning steps needed to mitigate the projected increase in cancer incidence and associated deficiencies in the cancer workforce. This paper reviews current efforts to track the oncology workforce and aims to link these efforts with the WHO global health workforce milestones 2020-2030 for achievement of the Sustainable Development Goals.
**Knowledge Generated**
We propose the establishment of cancer workforce registries using the *WHO Minimum Data Set for Health Workforce Registry* by adapting the data set to suit the multidisciplinary nature of the cancer workforce. This paper describes various stakeholders to be involved in this task.
**Relevance**
This paper is novel as it proposes establishing and implementing cancer workforce registries in all countries including low-middle–income countries. This is an essential step toward closing the gap between the rising incidence of cancer and the inadequate cancer workforce.

Global cancer control efforts are hampered by limitations in the available workforce; this is particularly evident in LMIC.^6-9^ A global analysis conducted by Union for International Cancer Control on national cancer control plans concluded that health workforce planning was inadequately addressed across cancer plans.^10^ The current literature focuses on workload and the shortage of the health care workforce, mostly among physicians, but has largely neglected the policy and planning required to address this shortage.

This paper aims to bridge the knowledge gap and provide a framework that can address the projected shortages in the oncology workforce. *Minimum Data Set for Health Workforce Registry* is a document published by the WHO in 2015 that outlines a tool to develop or modify an existing information system to document national health care workers.^11^ Establishing cancer workforce registries using this proposed framework is an essential step to close the gap between the rising incidence of cancer and the inadequate cancer workforce (Appendix Table A1).^5^

## POLICY PROPOSAL

Health workforce registries are an integral part of a health information system as they allow for better understanding and planning of health workforce demands and capacities.^11^ A registry will facilitate the monitoring of workforce patterns such as health care worker migration and changes in the flow of both the institutional and regional workforce supply/demand ratios. Most importantly, this allows policy makers and educators to consider training programs and educational capacity to meet their jurisdiction’s health care needs. The workforce registry can also promote an efficient hiring process, allowing the registry to function as a liaison between institutions, societies, and government bodies that are responsible for training and recruiting health care workers. Furthermore, it will be helpful in monitoring financial aspects of the health care workforce such as payroll, benefits, and performance management.^11^ Finally, a cancer workforce registry will provide the information required to efficiently allocate and prioritize both human and financial resources for evidence-based policy interventions.

The WHO global health workforce milestone for 2020 called for the establishment of health workforce registries, “All countries are making progress on health workforce registries to track health workforce stock, education, distribution, flows, demand, capacity and remuneration.”^5^ This lack of progress in health care workforce registries presents a further hindrance in meeting the 2030 UN SDGs.

## DETAILS OF THE POLICY

The *WHO Minimum Data Set for Health Workforce Registry* identified 10 essential items for health care workforce registry (Table 1).^11^ The registry can be divided into four functional domains corresponding to the health workforce continuum: pre-entry, entry, exist, and exit domains.^11^ The pre-entry domain (1) is associated with the planning aspect of the workforce registry including budget and workforce needs. The entry domain (2) deals with preparation of the workforce including education, regulation, and recruitment of the workforce. The exist domain (3) focuses on managing the workforce including services such as payroll, transfers, and disciplinary action. The exit domain (4) consists of the retiring workforce, which includes pensions, retirement, and discharge.

**TABLE 1:**
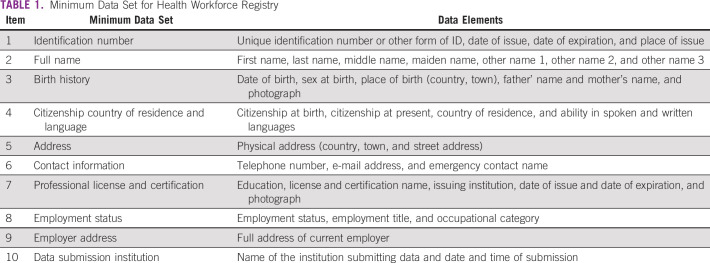
Minimum Data Set for Health Workforce Registry

The WHO Minimum Data Set hierarchically establishes competencies according to different institutional levels. Ministries will be responsible for establishing the cancer workforce registry.^11^ Teaching institutions that focus on health workforce production and training will submit data quarterly. Health professional registration and regulatory bodies will provide information regarding licensing and certification that will be submitted quarterly. Health workforce employers provide nominal roll or payroll data of active employees to be submitted monthly. Retirement administration can provide data on inactive health workforce to be submitted quarterly. This will ensure an updated, reliable, and consistent databank with cohesive data present.

## CURRENT METHODS TO TRACK THE CANCER WORKFORCE

A literature search was conducted in the following electronic databases: Medline, Embase, Google Scholar, and PubMed from inception to June 2019 using terms such as oncology, health workforce, and registr*. We also supplemented the search by reviewing bibliographies of articles that met the inclusion or search criteria to ensure that eligible studies not captured by the search strategy were included. Furthermore, a search for reports from the WHO and other professional oncology organizations was also conducted. However, there were not enough articles to screen from the electronic databases that met the selection criteria because of very limited research on this topic. Current methods of tracking the cancer workforce can be categorized as either surveys or workforce information systems (WISs). The findings are summarized in Table 2.^8,12-18,20-23^

**TABLE 2:**
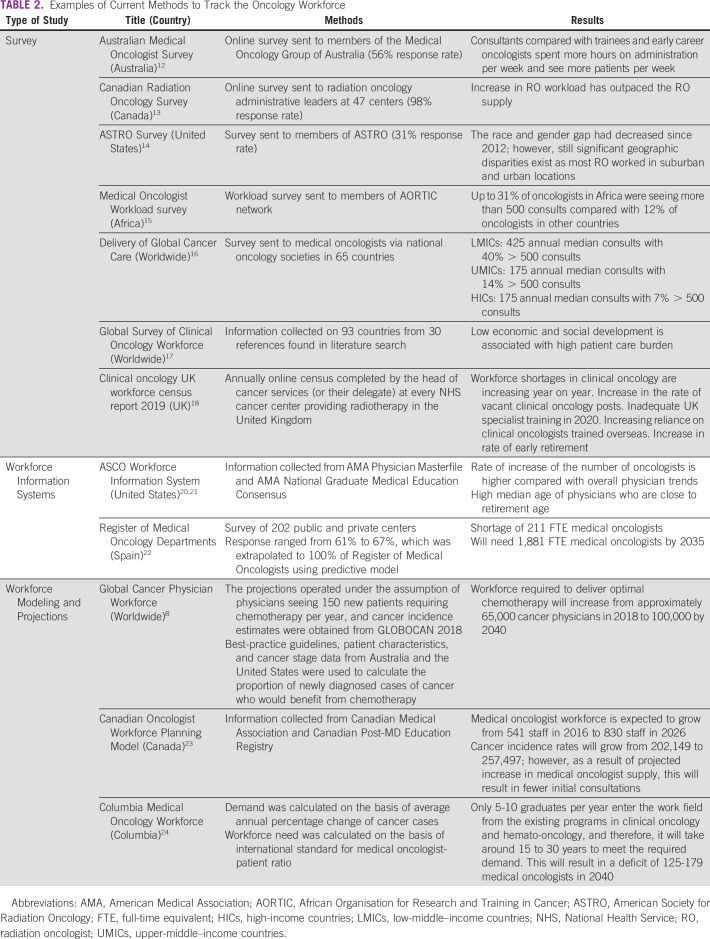
Examples of Current Methods to Track the Oncology Workforce

### Surveys

The Australian medical oncologist survey used an online self-administered questionnaire to collect demographic and work-related information from members of the Medical Oncology Group of Australia in 2016.^12^ The response rate was 56% (354 of 633), and it identified some key elements of the Australian medical oncology workforce. When compared with trainees and early career oncologists, consultants spent more hours on administration per week and saw more patients per week. The majority of medical oncologists worked in capital cities and metropolitan areas in the three most populated states.

The Canadian Association of Radiation Oncology sent an online survey to radiation oncologists’ administrative leaders at 47 Canadian cancer centers.^13^ The survey included questions about demographics, clinical workload, and equipment inventory from 2014 to 2016. The response rate was 98% (46 of 47 centers), and the survey concluded that the increase in radiation oncologist workload outpaced the radiation oncologist supply.

The American Society for Radiation Oncology (ASTRO) sent a workforce survey to 3,856 radiation oncologists in 2017 collecting information on work status, sex, US region, and race or ethnicity.^14^ The response rate was 31% (1,174 of 3,856). A notable finding was a decreased race and gender gap since 2012; yet there were still significant geographic disparities present as most radiation oncologists worked in suburban and urban locations.

A recent workload survey of African oncologists within the African Organisation for Research and Training in Cancer (AORTIC) network found that the median number of annual consults per oncologist is much higher in Africa compared with other countries (325 *v* 175).^15^ Up to 31% of oncologists in Africa were seeing more than 500 consults compared with 12% of oncologists in other countries. This survey further emphasized the need for governments to develop new methods to increase the capacity of cancer care systems across Africa and improve the oncologist-to-patient ratio.^15^

Furthermore, an online survey was distributed to medical oncologists through national oncology societies in 65 countries to investigate and compare current medical oncology workload on a global scale.^16^ A total of 1115 physicians completed the survey: 13% (147 participants) from LMICs, 17% (186) from upper-middle–income countries, and 70% (782) from high-income countries. In LMICs, the number of median annual consults seen by a medical oncologist was 425 with 40% seeing more than 500 consults.^16^ This was comparatively higher than upper-middle–income countries where medical oncologists saw 175 annual median consults with 14% seeing more than 500 consults. Finally, medical oncologists also had 175 annual median consults with 7% seeing more than 500 consults.^16^ An additional review by Mathew^17^ in 2018 found similar results when describing the availability of clinical oncologists from 93 countries across all continents and Sociodemographic Index classifications. In 22 countries (24%), a clinical oncologist cares for < 150 patients, all of which are classified as middle-high–income to high-income countries except China (middle). In 39 countries (Sociodemographic Index ranging from low to high), a clinical oncologist would care for > 500 patients with cancer. Finally, in 27 countries (low-middle–income and low-income countries except Sri Lanka and South Africa), one or zero clinical oncologists serve > 1,000 incident cancer cases.^17^ This pattern shows that a low economic and social development of a country is strongly associated with a shortage of human resources.^17^ These studies further emphasized the increasing cancer burden across the globe and the discrepancy present between different countries.

Since 2008, the Royal College of Radiologists (RCR) has gathered clinical oncology workforce data annually through an online census, which is completed by the head of cancer services (or their delegate) at every National Health Service cancer center providing radiotherapy in the United Kingdom.^18^ The 2019 census findings were thought to be concerning according to RCR. Workforce shortages in clinical oncology are increasing year on year. This is coupled with increase in the rate of vacant clinical oncology posts, a woefully inadequate UK specialist training in 2020, increasing reliance on clinical oncologists trained overseas, and increase in rate of early retirement.^18^ Several recommendations were made on the basis of the survey findings such as increasing training positions and streamlining pathways for recruiting skilled doctors from overseas.

The European Society of Medical Oncology is in the process of conducting a workforce survey in collaboration with WHO.^19^ The aforementioned studies further highlight the utility of an oncology workforce registry for making evidence-based decisions regarding the modification of the oncology workforce to address the increasing cancer burden.

### WISs

There are currently two published WISs. ASCO established a WIS to assemble data on oncologists and cancer incidence and prevalence.^20^ Information related to sex, international medical training, race, and ethnicity was collected using the American Medical Association (AMA) Physician Masterfile and AMA National Graduate Medical Education Consensus. ASCO acknowledged some limitations of this methodology, such as AMA Masterfile not being suitable for workforce analysis and the information for the Masterfile being outdated as the census is conducted every 3-4 years. However, the WIS made some significant conclusions about the oncology workforce in the United States in 2011 and again in 2018.^21^ The number of oncologists is increasing at a higher rate compared with overall physician trends. There are also a high proportion of international medical graduates on temporary training or study visas who may not permanently enter the workforce. Furthermore, physician median age is quite high (51 years in 2017), and the number of oncologists age 64 years or older is greater than those younger than age 40 years from 2009 to date, suggesting that a substantial proportion of the oncology workforce is nearing retirement. The percentage of oncologists who are female has increased from 24.2% in 2007 to 33.2% in 2017.^21^ Although Black people comprise 13% of the population in the United States, Black oncology fellows are still substantially under-represented, accounting for only 4.2% of oncology fellows.^21^ These observations based on WIS allow ASCO to evaluate workforce initiatives on the basis of trends and to address policy changes accordingly.

The Spanish Society of Medical Oncology established the Register of Medical Oncology Departments in 2014, which includes medical oncologists in both public and private hospitals in Spain.^22^ Data were collected from a survey of 202 public and private centers. Complete information was obtained from 67% of medical oncologists registered in public centers and from 61% of oncologists registered in private centers. The study found that there is a shortage of 211 full-time equivalent (FTE) medical oncologists and predicts that to maintain 158 new cases/FTE ratio, there need to be 1,881 FTE medical oncologists by 2035.

### Workforce Modeling and Projections

Workforce modeling and projections are valuable for predicting and planning workforce needs, as well as evidence for policy makers when budgeting human resource needs in cancer care as they estimate the gap between available service provision and demand for care. Primarily conducted by academic institutions, workforce models and projections can act as a reference point for institutional and governmental decision makers when developing cancer care control strategies.

A modeling study published in *The Lancet Oncology* estimated changes in the global cancer physician workforce required to deliver first-course chemotherapy from 2018 to 2040.^8^ Using best-practice guidelines, patient characteristics and cancer stage data from Australia and the United States were used to calculate the proportion of newly diagnosed cases of cancer who would benefit from chemotherapy. These rates were then applied to cancer incidence estimates obtained from GLOBOCAN 2018 and the projections operated under the assumption of physicians seeing 150 new patients requiring chemotherapy per year.^8^ The authors found that the workforce required to deliver optimal chemotherapy will increase from approximately 65,000 cancer physicians in 2018 to 100,000 by 2040.^8^

A Canadian Oncologist Workforce Planning Model was developed to predict medical oncologist supply and demand in 2026.^23^ Data from Canadian Medical Association and Canadian Post-MD Education Registry data were used to estimate the medical oncology supply, whereas the demand for medical oncologists was estimated using data from Canadian Cancer Statistics and Alberta Cancer Registry. On the basis of a forward calculation model, the medical oncologist workforce is expected to grow from 541 in 2016 to 830 in 2026. Cancer incidence rates will grow from 202,149 to 257,497. However, as a result of projected increase in medical oncologist supply, this will result in fewer initial consultations, which will decrease from an average of 168.5 consultations in 2016 to 129.2 consultations per medical oncologist in 2026.

An analysis of the current Colombian medical oncologist workforce published in 2019 concluded that the projected increase in the number of cases from 101,893/year in 2018 to 136,246/year in 2040 will result in a deficit of 125-179 medical oncologists.^24^ Only 5-10 graduates per year enter the work field from the existing programs in clinical oncology and hemato-oncology, which will take around 15-30 years to meet the required demand.

### Shortfalls of Current Methods of Tracking the Oncology Workforce

Most of the aforementioned studies were conducted in high-income countries by national cancer societies or academic institutions, rather than by Ministries of Health as recommended by the WHO. Most of the surveys and related efforts do not meet the WHO Minimum Data Set recommendations and WHO Health Care workforce milestones. There are some very promising results from ASCO WIS and The Spanish Register of Medical Oncology Departments. However, the two published information systems are limited to medical oncologists only. Surveys are limited by the fact that they constitute only a snapshot in time and some have low response rates. Some exceptions include the UK RCR annual census of clinical oncologists, which has a high response rate and provides consistent data annually. Limitation of modeling and workforce forecasts is that they need to be validated and rechecked for future accuracy on the basis of real-world data of the workforce. This will require a functional and reliable WIS.

Most surveys and WISs are centered on physician workforce mainly medical, radiation, or clinical oncologists. Cancer care is multidisciplinary, and the health workforce registries need to reflect this. This is especially important in cancer care as it requires a functional system that moves beyond oncologists and incorporates disciplines such as surgery, palliative care, nursing, pharmacy, etc. Without a cohesive system that includes all health care providers, patients will not receive the holistic care that is necessary in the fight against cancer. The existing and future WISs can be developed according to WHO Minimum Data Set, whereas other countries need to move past surveys and begin establishing workforce registries.

A limitation of our review of current workforce tracking in oncology is that some of the available systems or surveys might not be published in peer-review papers or might have not been captured by our current search methods.

## RELEVANCE TO LOW-MIDDLE–INCOME COUNTRIES

*The WHO Minimum Data Set for Health Workforce Registry* can serve as a useful tool for LMICs that are beginning to upscale their cancer workforce. This is an opportunity to establish and build on existing systems early in the process to collect workforce data that will allow for workforce policy decisions to be made in an evidence-based manner. For instance, India established the National Cancer Grid (NCG) in 2012 to link cancer centers across India.^25^ It aims to establish a standard of care across India and facilitate collaboration of research and exchange expertise. The NCG could benefit by adding a health workforce registry on the basis of the WHO Minimum Data Set guidelines to facilitate the NCG’s goals of a uniform standard of care. The number of cancer cases is projected to double in India by 2035, and a cancer workplace registry led by the NCG will allow for better planning and training of oncology health professionals.

## SUGGESTED FRAMEWORK AND CONCLUSION

**FIG 1:**
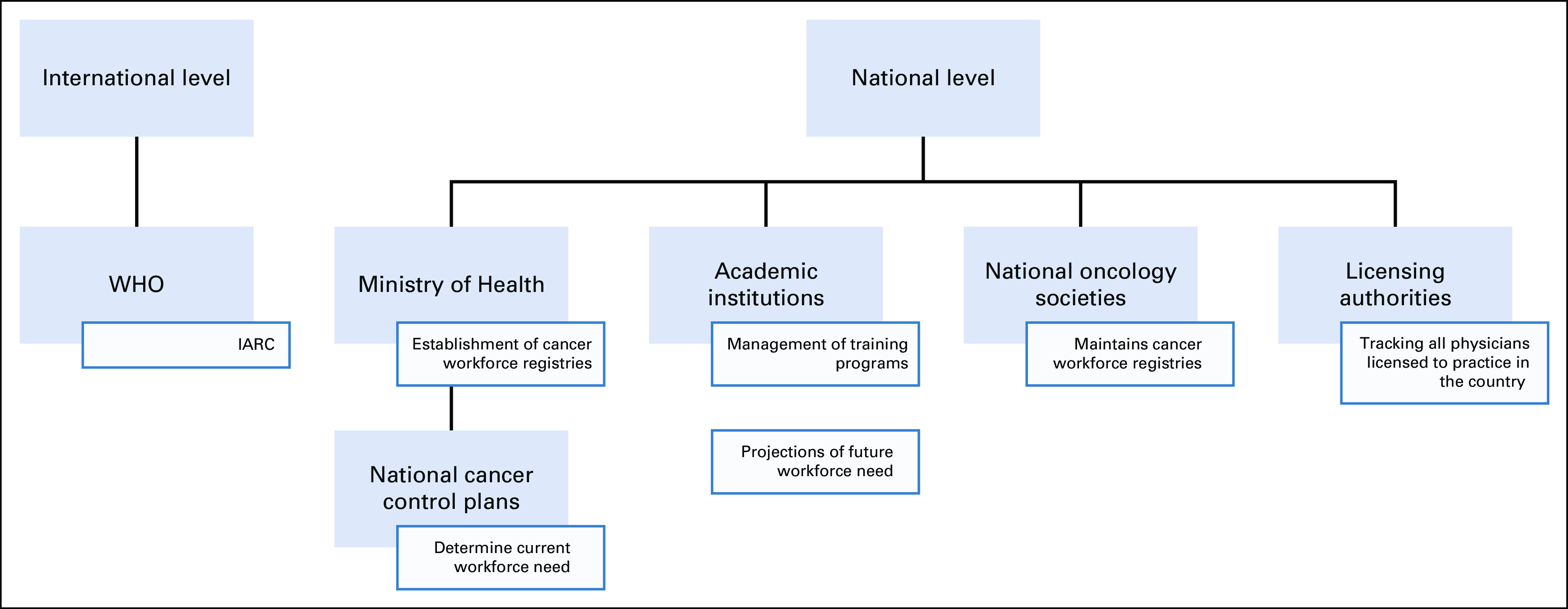
Proposed framework for cancer workforce registry. Framework of important actors and their respected responsibilities in the establishment and maintenance of a cancer workforce registry. IARC, International Agency for Research on Cancer.

According to *WHO Minimum Data Set for Health Workforce Registry*, Ministry of Health should be the designated body in charge of forming the registry. However, our review of current literature suggests that various individuals from academic institutions have taken the initiative to explore the current oncology workforce trends and model the future need on the basis of these trends. Our suggested framework (Fig 1) includes four key players, namely, academic institutions, national oncology societies, Ministries of Health (and other designated government institutions), and licensing authorities. Academic institutions in liaison with national oncology societies will continue to collect information regarding workforce trends using surveys and modeling data to predict future need based on the modeled data. In many cases, oncologists practice in private institutions and hospitals and are therefore not captured by academic institutions and national oncology societies. This is exemplified in a survey of 82 medical oncologists in India, where 48 (59%) practice exclusively in the private sector.^26^ To capture these numbers, licensing and accrediting authorities (eg, medical councils and colleges) need to be engaged as they can track all physicians licensed to practice in the country. This can be done across different oncology professions, as there is a dearth of evidence surrounding nursing and other allied health profession workforce in oncology. The Ministry of Health will be responsible for compiling the data obtained from various academic institutions and national oncology societies into a registry. National cancer control plans (developed by Ministries of Health) will help investigate and assess the growing need for the oncology workforce and inform future modeling.

Our review of the current status of efforts to meet the WHO guidelines for the implementation of oncology workforce registries demonstrates that the oncology community needs to develop and optimize methods to collect information for these registries. Adapting the *WHO Minimum Data Set for Health Workforce Registry* to suit the multidisciplinary nature of the cancer workforce would be an important step in obtaining the information required to make evidence-based policy decisions to address the anticipated oncology workforce shortages. Furthermore, demographic information is important as it can provide necessary information about equitable representation on the basis of sex and race. National cancer societies are likely to continue to lead such efforts, but ministries of health, licensing authorities, and academic institutions should contribute and collaborate in these efforts.

## Figures and Tables

**TABLE A1:**
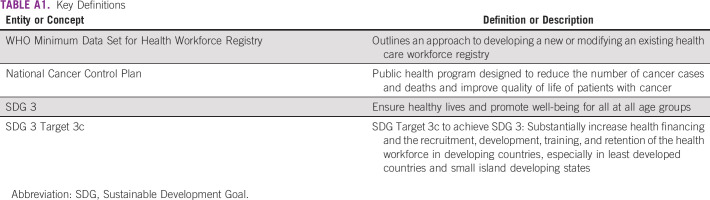
Key Definitions
